# Supplementary material for: Electronic Structure of NdFeCoB Oxide Magnetic Particles Studied by DFT Calculations and XPS
Source: Materials (Basel). 2023 Jan 29;16(3):1154. doi: 10.3390/ma16031154 (PMC9921898; doi:10.3390/ma16031154)
Supplement: Supplementary file 1 [file materials-16-01154-s001.zip › materials-2066333-supplementary.pdf]

Supplementary Materials

XRD charts

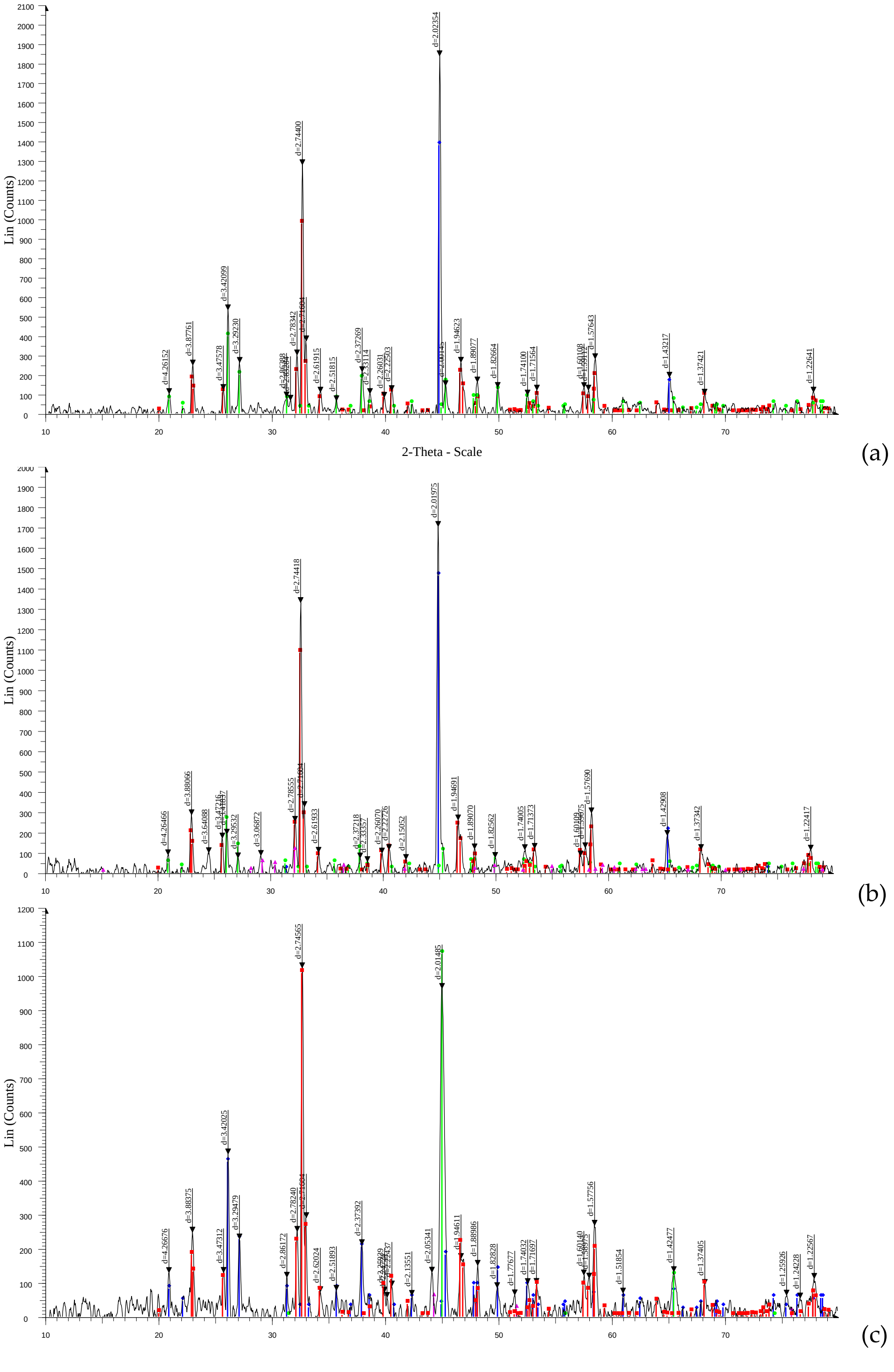

Figure S1. X-ray diffraction patterns of the $NdFe_{(1-x)}Co_xB$ oxide powders (a) x = 0, (b) x = 0.05, (c) x = 0.5.

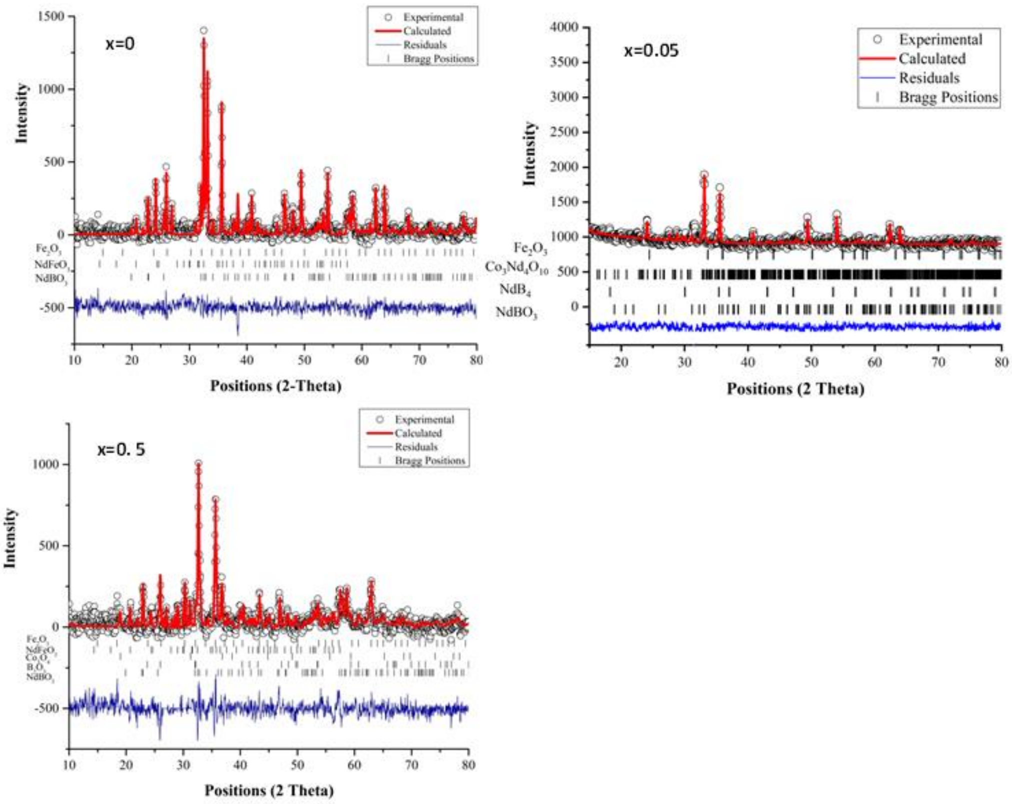

Figure S2. Rietveld plots of NdFe$_{(1-x)}$Co$_x$B oxide powders for x = 0, x = 0.05 and x = 0.50 along with the weight percentage of the phases.

Survey X-ray Spectra for concentration evaluation

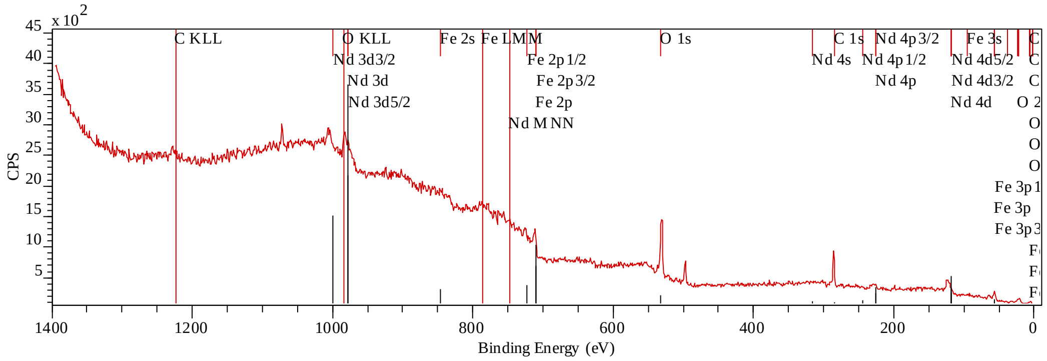

Figure S3. Overview spectrum of NdFe$_{(1-x)}$Co$_x$B oxide powders for x = 0.

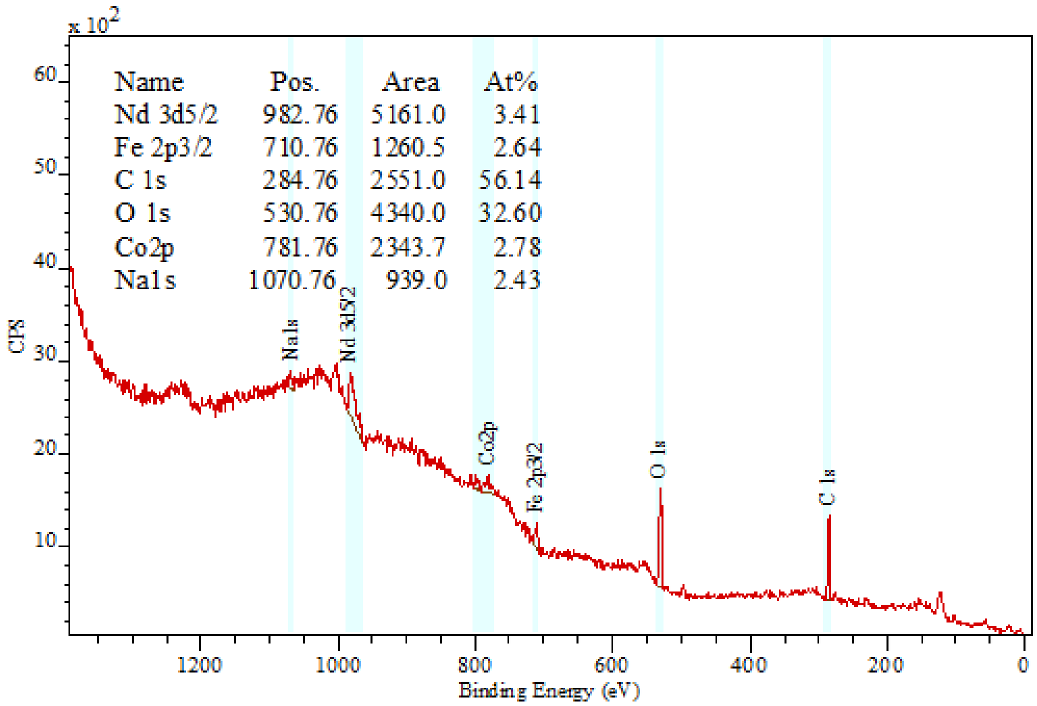

Figure S4. Overview spectrum of $NdFe_{(1-x)}Co_xB$ oxide powders for x = 0.05.

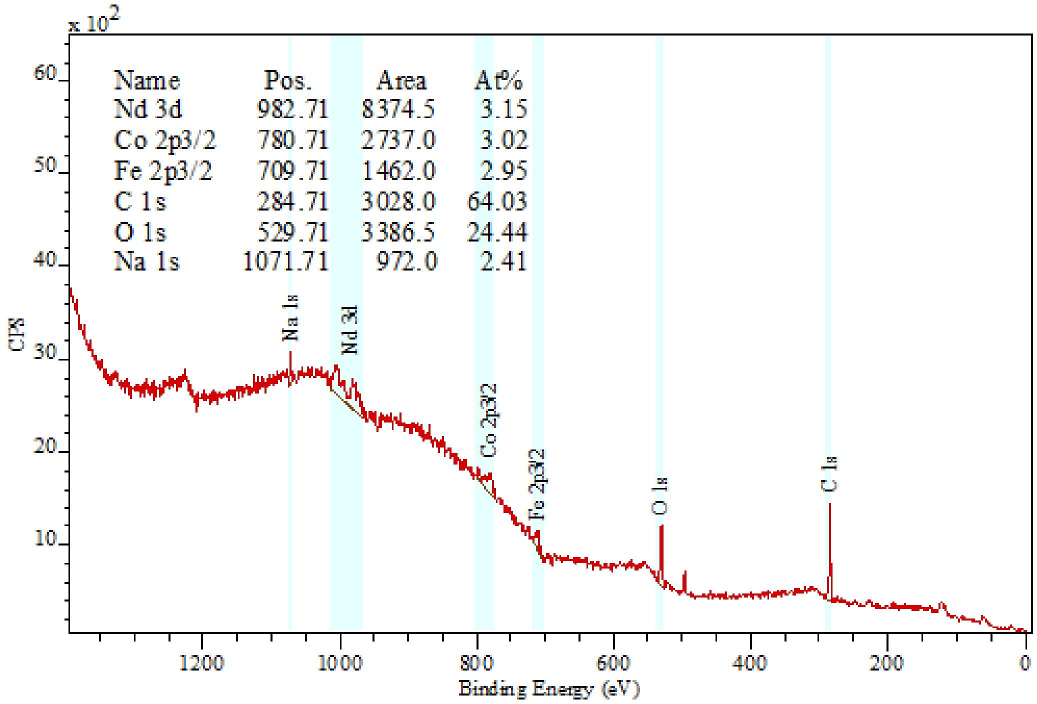

Figure S5. Overview spectrum of $NdFe_{(1-x)}Co_xB$ oxide powders for x = 0.5.
